# Identification of seasonal variation in the diagnosis of acute myeloid leukaemia: a population‐based study

**DOI:** 10.1111/bjh.18279

**Published:** 2022-05-31

**Authors:** Fernando Sánchez‐Vizcaíno, Carmen Tamayo, Fernando Ramos, Daniel Láinez‐González, Juana Serrano‐López, Raquel Barba, Maria Dolores Martin, Pilar Llamas, Juan Manuel Alonso‐Dominguez

**Affiliations:** ^1^ Bristol Veterinary School, Faculty of Health Sciences University of Bristol Bristol UK; ^2^ Department of Hematology Hospital Universitario de León León Spain; ^3^ Instituto Investigación Sanitaria FJD (IIS‐FJD) Madrid Spain; ^4^ Department of Internal Medicine Hospital Rey Juan Carlos Madrid Spain; ^5^ Department of Epidemiology Hospital Universitario Fundación Jiménez Díaz Madrid Spain; ^6^ Department of Hematology Hospital Universitario Fundación Jiménez Díaz Madrid Spain

**Keywords:** acute myeloid leukaemia, diagnosis, infection, leukaemias, seasonality

## Abstract

Until now, the role that seasonal factors play in the aetiology of acute myeloid leukaemia (AML) has been unclear. Demonstration of seasonality in AML diagnosis would provide supportive evidence of an underlying seasonal aetiology. To investigate the potential seasonal and long‐term trends in AML diagnosis in an overall population and in subgroups according to sex and age, we used population‐based data from a Spanish hospital discharge registry. We conducted a larger study than any to date of 26 472 cases of AML diagnosed in Spain between 2004 and 2015. Using multivariable Poisson generalized linear autoregressive moving average modelling, we found an upward long‐term trend, with monthly incidence rates of AML annually increasing by 0.4% [95% confidence interval (CI), 0.2%–0.6%; *p* = 0.0011]. January displayed the highest incidence rate of AML, with a minimum average difference of 7% when compared to February (95% CI, 2%–12%; *p* = 0.0143) and a maximum average difference of 16% compared to November (95% CI, 11%–21%; *p* < 0.0001) and August (95% CI, 10%–21%; *p* < 0.0001). Such seasonal effect was consistent among subgroups according to sex and age. Our finding that AML diagnosis is seasonal strongly implies that seasonal factors, such as infectious agents or environmental triggers, influence the development and/or proliferation of disease, pointing to prevention opportunities.

## INTRODUCTION

Acute myeloid leukaemia (AML) is a rare disease, yet responsible for a large number of cancer‐related deaths.^1^ The crude incidence rate of AML found in 44 European cancer registries in the years 2000–2002 was 3.6 [95% confidence interval (CI): 3.5–3.7] per 100 000 persons.^2^ AML is slightly more frequent among males and is the most common acute leukaemia in infants and adults, with the highest incidence rates being in older people.^1^, ^2^

Mutations that cause AML can occur due to an inherited mutant gene or exposition to certain carcinogens, such as chemotherapy, radiotherapy, ionizing radiation, tobacco and benzene.^3^, ^4^, ^5^, ^6^ However, the role of other environmental triggers and biological factors in tumour promotion and/or in proliferation of AML remains unknown.^7^

Demonstration of seasonal variation in the occurrence of AML would, firstly, provide supportive evidence of aetiology by seasonal factors, such as infectious agents or environmental factors; and, secondly, focus research onto the etiological role of such factors. The value of this approach has been recognized previously, although several early studies explored and failed to draw conclusive evidence of the potential seasonality of AML.^8^, ^9^, ^10^, ^11^, ^12^ A more recent study, however, detected a peak of adult AML diagnoses in the United States during December–January using a large sample of cases between 1992 and 2008.^13^ Results from this study have since not been confirmed or refuted in other populations.

Due to a lack of consensus on the possible seasonal variation in AML incidence and the progress made within the last decade in data recording and in the development of statistical models for analysing seasonal incidence data,^14^ further studies examining the possible temporal pattern in the presentation of AML are required. This study utilized population‐based data on cases of AML occurring in Spain from a nationwide hospital discharge registry for the years 2004–2015. This is, to our knowledge, the largest study aimed at investigating the potential seasonal and long‐term trends in AML incidence in an overall population and in subgroups according to sex and age, while employing novel statistical models with serial dependence for discrete‐valued time series.

## METHODS

The study was approved by the Ethics Committee for Clinical Research of Hospital Universitario Fundación Jiménez Díaz (EO 100/2017_FJD‐HRJC), in accordance with the Declaration of Helsinki. The study was registered on https://clinicaltrials.gov (Identifier: NCT03433521).

### Data collection and management

#### At‐risk population

The census of the Spanish general population was obtained from the Spanish National Statistics Institute for the period 2004–2015.^15^ Data were collected on an annual basis and were stratified by sex in the age groups: 0–4, 5–19, 20–49, 50–64, 65–74, and 75 years and over.

#### Study population

The *Conjunto Mínimo Básico de Datos* (CMBD) is a national hospital discharge database that contains anonymized data for approximately 98.0% of public hospital admissions in Spain, and it covers 99.5% of the Spanish population.^16^, ^17^

Data were obtained from the CMBD on every recorded case of AML [International Classification of Diseases (ICD)‐9 codes: 205.0, acute myeloid leukaemia; 205.3, myeloid sarcoma; 206.0, acute monocytic leukaemia; 207.0, acute erythraemia and erythroleukemia; and 207.2, megakaryocytic leukaemia] in Spain during the period 1997–2015. We extracted information from the register of each case about the date of admission, discharge date, anonymous identifier for each patient, ICD‐9 codes, sex and date of birth from which we derived age groups as described for the at‐risk population. ICD‐9 codes included a second decimal place indicating whether the cancer was active (.00), in remission (.01), or was considered a recurrence (.02). For patients hospitalized on more than one occasion, only the record corresponding to their first diagnosis of AML was selected. To reduce the likelihood of including patients who had been diagnosed with AML before 1997, only patients admitted to hospital and diagnosed with AML in or after 2004 were included in the analysis. Patients whose first recorded diagnosis of AML was classified as remission or recurrence (*N* = 3471) or that only had the first decimal place of the ICD‐9 code recorded (*N* = 16), were excluded.

### Statistical analysis

All analyses were done in R version 3.6.3.^18^ The date of hospital admission from which the diagnosis of AML was established is the date used for analysis (for simplicity hereafter 'date of diagnosis'). AML cases per month were standardized to months of equal length. Age/sex‐standardized monthly incidence rates of AML were calculated using the census of Spanish population in 2010 as a 'standard' population. Age‐standardized‐ and sex‐standardized monthly incidence rates of AML were calculated (2010 Spain standard population) and used in stratified analyses performed for sex and age groups respectively. Incidence rates are expressed as the number of AML cases per million person‐months at risk if not indicated otherwise.

Nine separate time‐series decompositions were performed as an initial exploratory analysis on the monthly incidence rates of AML using data for all cases, and data for each sex and age group. Time series were decomposed into trend, season and remainder components using the STL (Seasonal and Trend decomposition using Loess) method.^19^

Nine separate Poisson generalized linear autoregressive moving average (GLARMA) models were fitted to evaluate the temporal dynamics in AML incidence using data for all cases, and data for each sex and age group. A detailed description of GLARMA models can be found elsewhere,^14^ and an enhanced description of the specification of the models used is contained in the Appendix S1. Explanatory variables considered within each model included *trend* (modelled as a linear trend), *monthly seasonality* (included as a categorical variable with 12 levels) and *December 2015* (dummy variable with two levels). The rate of AML in December 2015 was identified as an outlier from the STL analysis. To account for such an outlier in the data and remove its effect, the variable *December 2015* was tested in each model. Multivariable models underwent manual backward step‐down selection to minimize the Akaike information criterion. The selection process of the appropriate lags for the autoregressive (AR) and moving average (MA) components is described in the Appendix S1. The best model was selected based on the likelihood ratio test (LRT) that serial dependence parameters are equal to zero. A version of the Fisher scoring iteration was used to locate a maximum likelihood fit for the model.^14^ If overdispersion was detected, then a negative binomial GLARMA model was fitted and retained as the final model if an LRT indicated significantly improved fit. Statistical significance was defined as *p* < 0.05.

## RESULTS

A total of 26 475 individual patients with a first diagnosis of active AML were hospitalized in Spain and registered at the CMBD during 2004–2015. Information on the sex of the patient was missing for three of the cases, leaving a total of 26 472 patients in the study population. The census of the Spanish population and stratified by sex and age for years 2004–2015 can be found as Table S1.

Descriptive characteristics of sex and age of the patients are shown in Table 1. A greater proportion of cases were male (56.0%) than female. The median age at diagnosis was 67 years (range: 0–103 years; interquartile range: 26 years).

**TABLE 1:** Acute myeloid leukaemia diagnoses in Spain by age and sex from 2004 to 2015

Age (years)	Number (%) of diagnoses by sex		Total (%)
	Female	Male	
0–4	189 (1.62)	195 (1.31)	384 (1.45)
5–19	360 (3.09)	464 (3.13)	824 (3.11)
20–49	2355 (20.23)	2645 (17.83)	5000 (18.89)
50–64	2451 (21.06)	3099 (20.90)	5550 (20.97)
65–74	2435 (20.92)	3770 (25.42)	6205 (23.44)
≥75	3851 (33.08)	4658 (31.41)	8509 (32.14)
Total (%)	11 641 (43.97)	14 831 (56.03)	26 472 (100)

The mean standardized yearly and monthly incidence rates of AML diagnoses over the study period were 47.62 cases per million person‐years (standard deviation: 1.01 cases per million person‐years) and 3.97 cases per million person‐months (standard deviation: 0.43 cases per million person‐months) respectively. A higher monthly incidence rate was observed in January in the overall population and in stratified analyses of sex and the age groups 5–19, 20–49, and 50–64 years (Table 2).

**TABLE 2:** Mean standardized incidence rates of acute myeloid leukaemia per year and calendar month, 2004–2015

Population	Standardization^a^ (2010 Spanish population)	Mean incidence rate per million person‐months												Mean incidence rate per million person‐years
		Jan	Feb	Mar	April	May	June	July	Aug	Sept	Oct	Nov	Dec	
All	Age/sex‐standardized incidence rates	4.45	4.14	4.02	3.88	3.99	4.01	3.94	3.77	3.95	3.99	3.73	3.74	47.62
Female	Age‐standardized incidence rates	3.61	3.51	3.28	3.26	3.37	3.07	3.17	3.16	3.14	3.21	3.08	2.99	38.85
Male	Age‐standardized incidence rates	5.53	5.02	4.98	4.74	4.79	5.28	4.95	4.61	4.98	5.00	4.60	4.73	59.23
0–4 years	Sex‐standardized incidence rates	1.57	1.01	1.32	0.91	0.60	1.10	1.60	1.31	1.44	0.97	1.18	1.04	14.07
5–19 years	Sex‐standardized incidence rates	1.06	0.81	0.90	0.91	0.76	0.74	0.85	0.66	0.75	0.94	0.83	0.96	10.18
20–49 years	Sex‐standardized incidence rates	2.01	1.74	1.64	1.71	1.58	1.54	1.49	1.53	1.65	1.69	1.62	1.54	19.74
50–64 years	Sex‐standardized incidence rates	5.46	4.94	5.08	4.64	5.25	4.57	4.98	4.38	4.77	4.84	4.50	4.37	57.81
65–74 years	Sex‐standardized incidence rates	12.38	12.41	10.89	10.39	11.67	11.99	10.97	10.02	11.11	11.06	9.86	10.55	133.23
≥75 years	Sex‐standardized incidence rates	16.55	16.23	16.04	15.77	15.43	17.43	16.08	16.43	15.71	15.94	15.16	15.32	192.09

^a^ Acute myeloid leukaemia cases per month were standardized to months of equal length.

**FIGURE 1:**
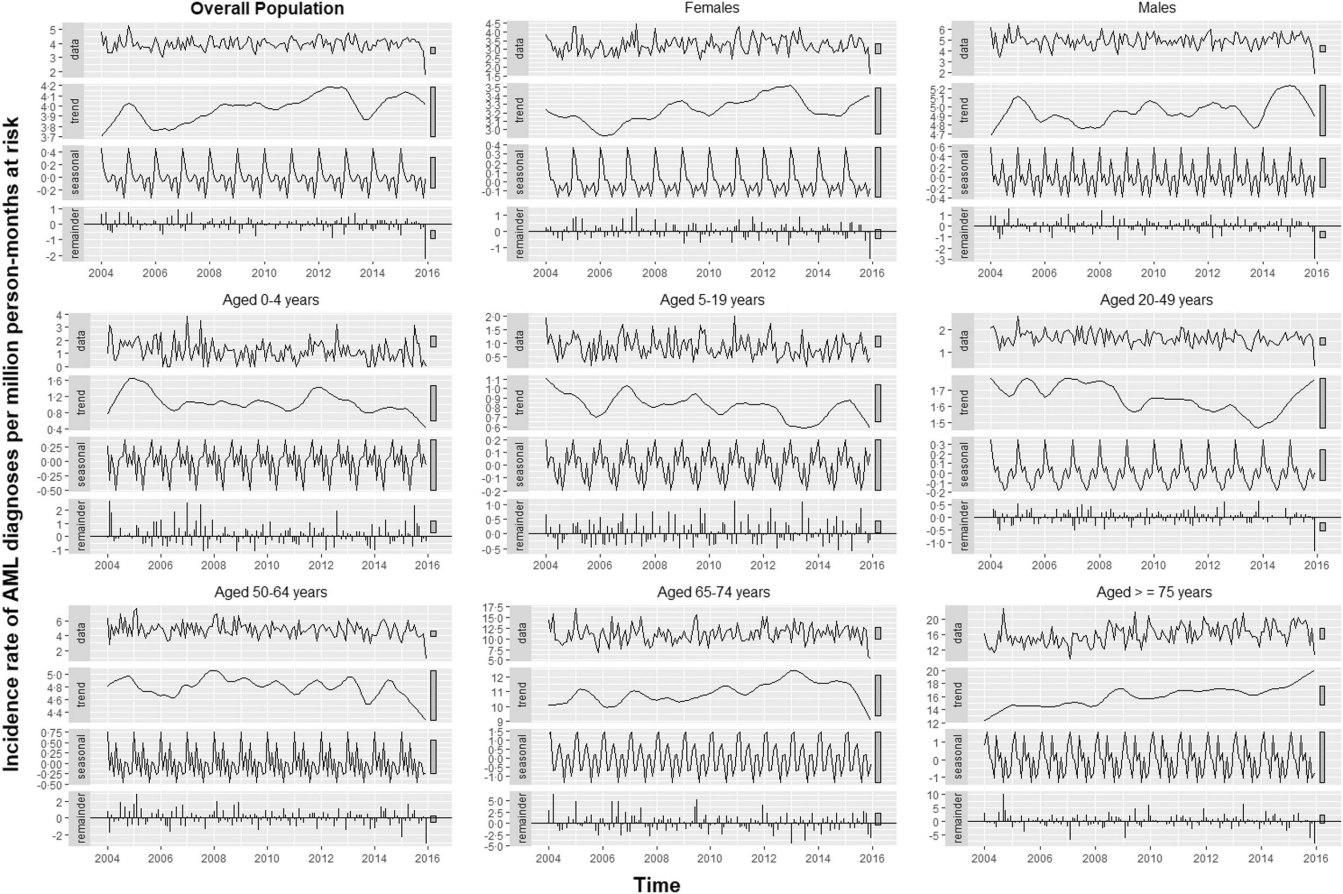
Decomposition of the standardized monthly incidence rates of AML diagnoses in Spain from 2004 to 2015. Nine separate time‐series decompositions are depicted using data for all cases (panel titled 'Overall Population') and for cases stratified by sex and age. Each panel includes the observed series (named 'data') and its three additive components (i.e. trend, seasonal and remainder) obtained from a robust STL (Seasonal and Trend decomposition using Loess) decomposition with flexible trend and fixed seasonality. The grey bars to the right of each panel show the relative scales of the components. Each grey bar represents the same length but because the plots are on different scales, the bars vary in size.

Nine time‐series plots depicting standardized monthly incidence rates of AML diagnoses for the overall population and stratified by sex and age along with their three components are shown in Figure 1. STL decomposition of the incidence rates observed in the overall population exhibited seasonal fluctuation with a peak in January (Figure 1; Overall population — seasonal panel). A slight upward trend was apparent from visual inspection with an up‐turn in early 2005 and a down‐turn at the end of 2013 (Figure 1; Overall Population — trend panel). The decomposition was characterized by a strong remainder component (Figure 1; Overall population — remainder panel) that explains most of the variability (72.3%) of the original signal (Table S2). The seasonal component displayed a higher amplitude than the trend component. The seasonal and trend components represented 18.1% and 8.0% of the variability of the original signal respectively (Table S2). In stratified analyses, STL decompositions provided a variety of trend, seasonal and remainder components in terms of shape, amplitude (Figure 1) and variability (Table S2). The seasonal component exhibited an annual peak in January among females, males and age groups 5–19, 20–49, and 50–64 years (Figure 1).

**FIGURE 2:**
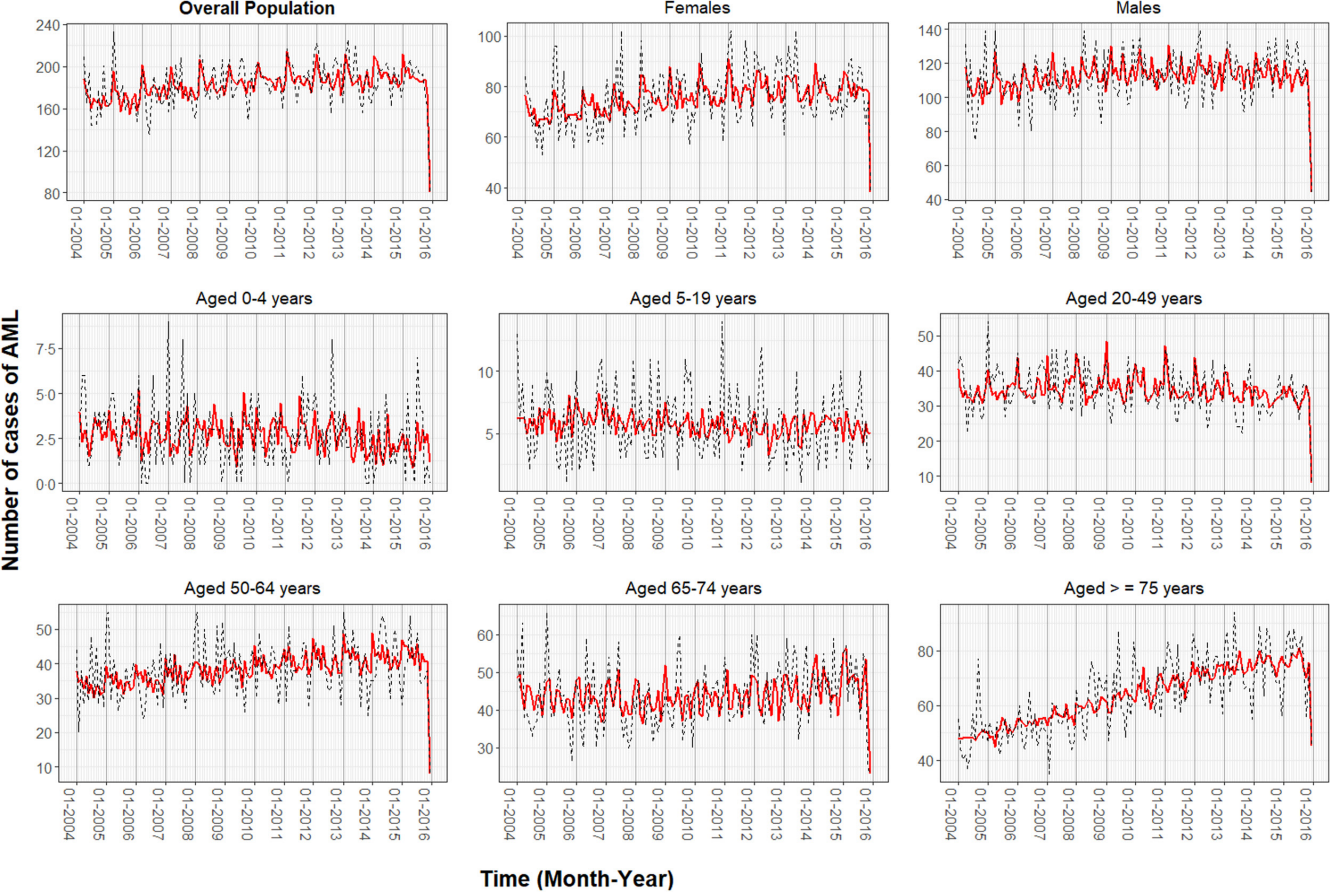
Fitted values from each final Poisson generalized linear autoregressive moving average (GLARMA) model. Time‐series plots depict observed counts (black dashed line) of monthly cases of acute myeloid leukaemia (AML) and predicted counts (red smooth line) of monthly AML cases using GLARMA for the overall population in Spain and stratified by sex and age from 2004 to 2015.

A graphical representation of the fitted values from each of the nine final Poisson GLARMA models along with the actual number of cases over time is depicted in Figure 2; it shows that, overall, the models' fitted values for the response closely trace the observed counts. The lags selected for the autoregressive and moving average components in each model are shown in Table S3. The autocorrelation functions of the Pearson's residuals indicate that the models have dealt adequately with any serial correlation present (Figure S1). Summary statistics of the final Poisson GLARMA model for all AML cases are available in Table 3. The final model included an upward linear long‐term trend, as well as the variables *monthly seasonality* and *December 2015*. The estimated monthly long‐term trend implies that the monthly incidence rates of AML diagnoses annually increased by 0.4% (95% CI, 0.2%–0.6%; *p* = 0.0011), given that the other covariates are held constant. January displayed the highest incidence rate of AML, with a minimum average difference of 7% when compared to February (95% CI, 2%–12%; *p* = 0.0143) and a maximum average difference of 16% compared to November (95% CI, 11%–21%; *p* < 0.0001) and August (95% CI, 10%–21%; *p* < 0.0001). The incidence rate of AML for December 2015 was 0.43 (95% CI, 0.34–0.54; *p* < 0.0001) times the average incidence rate for the rest of the study period. Further results from the final Poisson GLARMA models for each sex can be seen in Table 3, and for each age group in Table 4.

**TABLE 3:** Parameter estimates from the final Poisson GLARMA models fitted for the overall population and each sex

Parameter	Overall population				Females				Males			
	Estimates	SE	IRR (95% CI)	*p*	Estimates	SE	IRR (95% CI)	*p*	Estimates	SE	IRR (95% CI)	*p*
GLM coefficients												
Intercept	1.47	0.02	4.35 (4.17–4.54)	**<0.0001**	1.25	0.03	3.49 (3.26–3.74)	**<0.0001**	1.71	0.03	5.51 (5.23–5.80)	**<0.0001**
Trend	0.0003	0.0001	1.004^a^ (1.002–1.006)^a^	**0.0011**	0.0006	0.0002	1.007^a^ (1.002–1.012)^a^	**0.0061**	…	…	…	…
December 2015				**<0.0001**				**<0.0001**				**<0.0001**
No	…	…	1 (ref)	…	…	…	1 (ref)	…	…	…	1 (ref)	…
Yes	−0.84	0.11	0.43 (0.34–0.54)		−0.78	0.17	0.46 (0.33–0.64)		−0.91	0.15	0.40 (0.30–0.54)	
Month				**<0.0001**				**0.0019**				**<0.0001**
January	…	…	1 (ref)	…	…	…	1 (ref)	…	…	…	1 (ref)	…
February	−0.07	0.03	0.93 (0.88–0.98)		−0.05	0.05	0.95 (0.86–1.05)		−0.10	0.04	0.91 (0.84–0.98)	
March	−0.10	0.03	0.90 (0.85–0.96)		−0.11	0.05	0.89 (0.81–0.98)		−0.10	0.04	0.90 (0.84–0.97)	
April	−0.14	0.03	0.87 (0.82–0.93)		−0.11	0.05	0.90 (0.82–0.98)		−0.15	0.04	0.86 (0.79–0.93)	
May	−0.11	0.03	0.90 (0.84–0.95)		−0.07	0.05	0.93 (0.85–1.02)		−0.14	0.04	0.87 (0.81–0.94)	
June	−0.11	0.03	0.90 (0.84–0.96)		−0.17	0.05	0.84 (0.77–0.92)		−0.05	0.04	0.95 (0.88–1.03)	
July	−0.12	0.03	0.88 (0.83.94)		−0.13	0.05	0.87 (0.80–0.96)		−0.10	0.04	0.90 (0.84–0.97)	
August	−0.17	0.03	0.84 (0.79–0.90)		−0.14	0.05	0.87 (0.79–0.95)		−0.18	0.04	0.84 (0.77–0.91)	
September	−0.12	0.03	0.89 (0.84–0.94)		−0.14	0.05	0.87 (0.79–0.95)		−0.10	0.04	0.91 (0.84–0.98)	
October	−0.11	0.03	0.89 (0.84–0.95)		−0.13	0.05	0.88 (0.80–0.96)		−0.09	0.04	0.91 (0.84–0.99)	
November	−0.18	0.03	0.84 (0.79–0.89)		−0.16	0.05	0.85 (0.77–0.93)		−0.18	0.04	0.84 (0.77–0.90)	
December	−0.13	0.03	0.88 (0.83–0.93)		−0.14	0.05	0.87 (0.78–0.96)		−0.11	0.04	0.90 (0.83–0.97)	
ARMA coefficients												
Phi	−0.01	0.005	…	**0.0221**	…	…	…	…	−0.02	0.01	…	**0.0274**
Theta	−0.01	0.005	…	**0.0099**	−0.03	0.01	…	**<0.0040**	−0.02	0.01	…	**0.0132**

*Note*: (…) It indicates the reference level of each categorical variable included in the model and each non‐significant covariate which was not included in the model.Significant (*p* < 0.05) results are displayed in bold.

Abbreviations: CI, confidence interval; GLARMA, generalized linear autoregressive moving average; GLM, generalized linear model; SE, standard error.

^a^ For the trend, the coefficient estimates are per month, but the incidence rate ratios are annualized for clarity.

**TABLE 4:** Parameter estimates from the final Poisson GLARMA models fitted for six different age groups

Param	0–4 years				5–19 years				20–49 years				50–64 years				65–74 years				≥75 years			
	Est	SE	IRR (95% CI)	*p*	Est	SE	IRR (95% CI)	*p*	Est	SE	IRR (95% CI)	*p*	Est	SE	IRR (95% CI)	*p*	Est	SE	IRR (95% CI)	*p*	Est	SE	IRR (95% CI)	*p*
GLM coefficients																								
Intercept	0.70	0.11	2.02 (1.63–2.51)	**<0.0001**	−0.05	0.03	0.95 (0.89–1.02)	0.1703	0.69	0.05	1.98 (1.81–2.18)	**<0.0001**	1.69	0.04	5.42 (4.97–5.91)	**<0.0001**	2.51	0.04	12.3 (11.3–13.3)	**<0.0001**	2.65	0.02	14.2 (13.6–14.9)	**<0.0001**
Trend	−0.004	0.001	0.96^a^ (0.93‐0.98)^a^	**0.0007**	−0.002	<0.01	0.98^a^ (0.97‐0.99)^a^	**0.0001**	…	…	…	…	…	…	…	…	…	…	…	…	0.002	<0.01	1.02^a^ (1.01‐1.03)^a^	**<0.0001**
Dec 2015	…	…	…	…	…	…	…	…				**<0.0001**				**<0.0001**				**0.0015**				**0.0004**
No									…	…	1 (ref)	…	…	…	1 (ref)	**…**	…	…	1 (ref)	**…**	…	…	1 (ref)	**…**
Yes									−1.42	0.36	0.24 (0.12–0.49)		−1.66	0.36	0.19 (0.09–0.38)		−0.68	0.21	0.51 (0.33–0.77)		−0.54	0.15	0.58 (0.43–0.79)	
Month				**0.0007**	…	…	…	…				**<0.0001**				**0.0193**				**0.0006**	…	…	…	…
Jan	…	…	1 (ref)						…	…	1 (ref)	…	…	…	1 (ref)	**…**	…	…	1 (ref)	**…**				
Feb	−0.53	0.17	0.59 (0.42–0.81)						−0.14	0.07	0.87 (0.76–0.99)		−0.09	0.06	0.91 (0.80–1.04)		0.02	0.06	1.02 (0.91–1.15)					
Mar	−0.28	0.15	0.75 (0.56–1.002)						−0.21	0.07	0.81 (0.70–0.92)		−0.06	0.06	0.94 (0.84–1.06)		−0.12	0.06	0.89 (0.79–1.002)					
Apr	−0.54	0.16	0.58 (0.42–0.80)						−0.16	0.07	0.85 (0.74–0.97)		−0.14	0.06	0.87 (0.76–0.99)		−0.19	0.07	0.83 (0.73–0.95)					
May	−0.97	0.21	0.38 (0.25–0.57)						−0.23	0.07	0.80 (0.69–0.91)		−0.04	0.06	0.96 (0.85–1.09)		−0.04	0.06	0.96 (0.85–1.08)					
Jun	−0.38	0.15	0.68 (0.50–0.92)						−0.23	0.06	0.80 (0.71–0.90)		−0.18	0.07	0.83 (0.73–0.95)		−0.05	0.05	0.95 (0.85–1.06)					
Jul	−0.07	0.13	0.94 (0.72–1.22)						−0.28	0.05	0.75 (0.68–0.84)		−0.10	0.06	0.90 (0.79–1.02)		−0.12	0.06	0.88 (0.78–0.99)					
Aug	−0.19	0.14	0.82 (0.62–1.09)						−0.26	0.06	0.77 (0.68–0.87)		−0.23	0.07	0.80 (0.70–0.91)		−0.19	0.06	0.83 (0.74–0.92)					
Sep	−0.10	0.13	0.90 (0.69–1.17)						−0.19	0.07	0.83 (0.72–0.95)		−0.13	0.06	0.88 (0.77–0.99)		−0.10	0.06	0.90 (0.80–1.02)					
Oct	−0.47	0.16	0.62 (0.45–0.86)						−0.18	0.07	0.84 (0.73–0.96)		−0.12	0.06	0.89 (0.78–1.01)		−0.10	0.07	0.90 (0.79–1.03)					
Nov	−0.30	0.15	0.74 (0.55–0.99)						−0.20	0.07	0.82 (0.71–0.94)		−0.20	0.06	0.82 (0.73–0.92)		−0.23	0.06	0.80 (0.70–0.90)					
Dec	−0.40	0.16	0.67 (0.49–0.91)						−0.19	0.07	0.83 (0.72–0.95)		−0.17	0.07	0.85 (0.74–0.96)		−0.10	0.06	0.91 (0.80–1.03)					
ARMA coefficients																								
Phi	…	…	…	…	−0.08	0.03	…	**0.0145**	0.06	0.02	…	**0.0017**	0.03	0.01	…	**0.0160**	…	…	…	…	−0.02	0.009	…	**0.0121**
Theta^b^	−0.25	0.06	…	**<0.0001**	−0.08	0.03	…	**0.0159**	0.04	0.02	…	**0.0093**	…	…	…	…	0.03	0.01	…	**0.0114**	0.02	0.01	…	**0.0107**
Theta^c^	…	…	…	…	−0.10	0.03	…	**0.0018**	…	…	…	…	…	…	…	…	−0.03	0.01	…	**0.0167**	…	…	…	…

*Note*: (…) indicates the reference level of each categorical variable included in the model and each non‐significant covariate which was not included in the model. Significant (*p* < 0.05) results are displayed in bold.

Abbreviations: CI, confidence interval; Est, estimates; GLARMA, generalized linear autoregressive moving average; GLM, generalized linear model; Param, parameter; SE, Standard error.

^a^ For the trend, the coefficient estimates are per‐month, but the incidence rate ratios are annualized for clarity.

^b^ Theta coefficients for models with only one lag and theta coefficients corresponding to the lowest lag in models with two lags (the selected lags for each model are shown in Table S3).

^c^ Theta coefficients corresponding to the highest lag in models with two lags (the selected lags for each model are shown in Table S3).

## DISCUSSION

Until now, the role that seasonal factors play in the aetiology of AML has been unclear.^20^ The demonstration of seasonality in AML diagnosis would provide supportive evidence of an underlying seasonal aetiology. This finding would allow further studies to focus on a detailed investigation of specific hypotheses relating to the etiological role of specific seasonal risk factors, such as infectious agents, allergens, or sunlight exposure. Using data from a population‐based hospital discharge registry we have carried out a larger study than any to date to investigate whether seasonal variation exists in the diagnosis dates of patients with AML. In addition, we evaluated potential long‐term trends in AML incidence and described the standardized incidence rates of AML diagnoses in Spain from 2004 to 2015.

The observed age/sex‐standardized monthly incidence rates of AML in Spain over the study period were in line with the age‐standardized monthly incidence rates found in a comparable study in the United States.^13^ Consistent with previous studies, AML incidence had a male predominance and increased progressively from later adulthood.^1^, ^2^, ^13^, ^21^, ^22^

This study showed a clear seasonal pattern in the diagnosis of AML, with the highest incidence rates of diagnosis observed in January. These findings are broadly similar to those in a large US‐based study by Calip et al. who found a peak of adult AML diagnoses during December–January from 1992 to 2008.^13^ Both studies utilize population‐based data; however, the U.S. study is restricted to adults aged 25 years and older from a limited number of locations, whilst our study includes data for all ages and from a registry with national coverage. Results from our study (*N* = 26 472 AML cases) and that by Calip et al.^13^ (*N* = 21 570) which analysed, respectively, the largest series of AML cases to date in Europe and the United States, provide strong evidence of a monthly variation in AML diagnosis and suggest a seasonal peak in winter months; especially in January, when the peak months found in both studies overlapped.

In contrast, however, earlier studies using cancer registry data from the United States and Europe have failed to find definitive evidence of seasonality in AML diagnosis.^9^, ^10^, ^11^, ^12^ There are a number of possible reasons why inconsistent results have occurred. Some previous studies have been based on a small number of cases and lacked statistical power.^8^, ^10^ Further, in early AML studies, where digitized records were non‐existent, it is likely that the main results were influenced by a low level of completeness and accuracy.^8^ Some of the contrasting results may also be due to the use of different and less advanced statistical approaches,^11^ or the failure of some studies to account for serial correlation and/or temporal trends.^9^

In stratified analysis, we observed the presence of seasonal variation in the incidence of AML among females and males with a peak in January. This is broadly consistent with previous studies elsewhere.^9^, ^13^ It is also possible that seasonality is more pronounced within particular age groups. Indeed, we found a significant seasonal effect for patients aged 0–4 years, whereas no such effect was detected among patients within the age group of 5–19 years. Interestingly, evidence of seasonal variation was also present in the remaining age groups, except for the oldest group. Elderly patients are frequently diagnosed with AML with myelodysplasia‐related changes, whilst younger patients are more commonly diagnosed with AML with recurrent genetic abnormalities.^23^ Such variations in the molecular background of AML between the older and younger AML cases may explain the disappearance of seasonal variation in the oldest group.

This study found strong evidence of seasonal variation in the diagnosis of AML, which suggests that seasonal risk factors, such as infectious agents or environmental factors, influence the development and/or proliferation of AML. Our results exhibited a significant annual peak in January. While the underlying reason for this result is unknown, it is possible that the observed peak in monthly AML risk coincides with seasonal elevations in the rates of infectious disease processes which are capable of precipitating or accelerating the course of AML. Indeed, infection has long been suspected as a possible factor in the aetiology of leukaemias.^24^, ^25^ Although no specific agent has yet been identified, parvovirus B19 is suggested to play a role in the aetiology of acute lymphoblastic leukaemia (ALL).^26^ Evidence for the involvement of infections in other haematological malignancies is more direct. Notably, a viral aetiology has already been demonstrated in adult T‐cell leukaemia/lymphoma related to HTLV‐1 infection,^23^ and Epstein–Barr virus appears to be aetiologically linked to Hodgkin lymphoma.^27^ In addition, several studies conducted in various countries have suggested a seasonal variation in the presentation and diagnosis of ALL and lymphomas which provides further support for an infectious aetiology for these diseases.^10^, ^12^, ^28^, ^29^, ^30^, ^31^ While ALL, lymphomas and AML are biologically different diseases and may have distinct aetiologies, there is no reason to think that an infectious component must only be limited to the first two. One reasoning behind such seasonality in AML diagnosis is that a seasonal infectious agent can be capable of promoting leukemogenesis.^28^ This explanation requires a short latency period between exposure to this agent and diagnosis of AML which is consistent with the Greaves model.^32^ However, cancers known to be associated with infection have been considered as resulting from the prolonged latency that occurs in chronic viral infections.^31^ An alternative explanation is that seasonally occurring infectious diseases can stimulate rapid proliferation of pre‐existent mutated and quiescent leukaemic stem cells, thereby hastening the diagnosis. This increase in leukaemic stem cell replication could be a consequence of a direct viral insertion in the genome of such cells or the result of an immunological response to an infectious agent.^33^, ^34^

It is also conceivable that the annual peak observed in January reflects an increased diagnostic activity during winter months; higher levels of healthcare demand because of winter‐related illness may lead to the diagnosis of AML. Note, however, that the incidence rates recorded in February and March were higher than the incidence rates observed in December. According to such an explanation, one expects to find higher incidence rates in December if cases that would have otherwise been diagnosed in February and March were being detected in the two months prior because of an increased diagnostic activity. Conversely, some have suggested that observed seasonal variation in certain cancers is likely to reflect the reduced diagnostic activity during vacation months.^35^ If such surveillance bias were present in our study, the peak found in January could reflect patients' delay in seeking medical care or health check‐ups until after Christmas holidays. We cannot discard an underestimate of the actual December AML incidence and a corresponding overestimate of the January incidence due to this phenomenon. However, it appears unlikely that the seasonal pattern observed in AML diagnoses can be only explained by lower diagnostic intensity during Christmas holidays, both because of the rapidity and severity of disease onset which will probably lead patients to immediately seek medical care and because of a lack of similar pronounced peaks after other vacation months such as the long summer holidays.

Because the data are from a population‐based registry that had a high level of completeness and accuracy throughout the study period, the main results are unlikely to have been influenced by ascertainment or recording bias. However, several potential limitations of this study need to be considered. One limitation is that AML cases were obtained from the CMBD registry as defined by ICD‐9 and no other AML classifications were available. Hence, further studies using more modern classifications of AML like the World Health Organization classification^23^ would be required to investigate the seasonal variation in the diagnosis of AML subtypes. Another limitation is that information on the date of onset of clinical symptoms was not available for analysis. However, modest variation in the lag period between first symptom and clinical diagnosis would generally be expected because of the rapid progress of the disease. A further limitation relates to the source of our data. Older patients with AML are more likely to be deemed 'unfit' or ineligible for intensive chemotherapy and instead they are frequently offered best supportive care or hypomethylating agents for which they often do not require hospitalization.^36^ This may have led us to underestimate the incidence rates of AML in older patients as only hospitalized patients were captured in our study. It is also of note that the incidence rate of AML in December 2015 was unusually low. Exploration of this outlier suggested that it can be explained by some patients being hospitalized in December 2015 who were discharged beyond year 2015 and therefore were not captured by our study. Data for each case were only available to us where the discharge date for a patient occurred within the study period from 1997 to 2015. We took account of such an outlier in the data by including December 2015 as an explanatory variable in each of the models.

In conclusion, this large study provides the strongest evidence of a seasonal variation in the diagnosis of AML, with a significant peak during the month of January. Such seasonal effect was consistent among subgroups according to sex and age. These findings could be used to raise the level of AML suspicion among haematologists where a cytopenia is detected in a complete blood count during winter months, particularly in January. Taken together, previous smaller studies have provided conflicting evidence, likely due to lower power and/or to the use of less advanced statistical approaches. Most studies assessing seasonal trends in AML diagnosis do so in northern‐hemisphere countries with temperate climates. Investigation of seasonality in cohorts from a wide range of geographical areas would be informative to determine the influence of latitude and climate on AML incidence. The demonstration of seasonality in our study strongly implies that seasonal factors, such as infectious agents or environmental triggers, influence the development and/or proliferation of AML, pointing to prevention opportunities. Analyses were stratified by important person‐level covariates such as sex and age that may confound seasonal trends in diagnoses. However, future studies that include more explanatory variables at individual level such as comorbid conditions and environmental exposure would augment the current results, providing further understanding into seasonal variation of AML diagnoses and the aetiology behind the observed variation.

## AUTHOR CONTRIBUTIONS

The study was conceived and designed by Juan Manuel Alonso‐Domínguez and Fernando Sánchez‐Vizcaíno. The funding for the project leading to this publication was acquired by Juan Manuel Alonso‐Domínguez and Fernando Sánchez‐Vizcaíno. The data were acquired by Raquel Barba, Maria Dolores Martin and Juan Manuel Alonso‐Domínguez. The data curation was carried out by Fernando Sánchez‐Vizcaíno and Carmen Tamayo. The methodology, formal analysis and visualization were conducted by Fernando Sánchez‐Vizcaíno. The manuscript was drafted by Fernando Sánchez‐Vizcaíno and Juan Manuel Alonso‐Domínguez. The manuscript was revised critically for important intellectual content by Carmen Tamayo, Fernando Ramos, Daniel Láinez‐González, Juana Serrano‐López, Raquel Barba, Maria Dolores Martin and Pilar Llamas. All authors gave final approval for publication.

## FUNDING INFORMATION

This study was supported by research funding from Celgene S.L.

## CONFLICT OF INTERESTS

Juan Manuel Alonso‐Dominguez received research funding from Incyte Corporation, Pfizer International, and Astellas Pharma Inc. outside the present work.

## Supporting information


Appendix S1

## Data Availability

Publicly available data were obtained from the Spanish National Statistics Institute.^15^ All other data and codes are available upon request from the authors after appropriate approvals.
