# Supplementary material for: Identification of seasonal variation in the diagnosis of acute myeloid leukaemia: a population‐based study
Source: Br J Haematol. 2022 May 31;198(3):545–55. doi: 10.1111/bjh.18279 (PMC9542150; doi:10.1111/bjh.18279)

**Identification of seasonal variation in the diagnosis of acute myeloid leukemia: a population-based study**

Fernando Sánchez-Vizcaíno^1*^, Carmen Tamayo^1^, Fernando Ramos^2^, Daniel Láinez-González^3^, Juana Serrano-López^3^, Raquel Barba^4^, Maria Dolores Martin^5^, Pilar Llamas^3,6^, Juan Manuel Alonso-Dominguez^3,6*^

^1^ *Bristol Veterinary School, Faculty of Health Sciences, University of Bristol, Langford Campus, BS40 5DU, United Kingdom*

^2^ *Department of Hematology, Hospital Universitario de León, León, Spain*

^3^ *Instituto Investigación Sanitaria FJD (IIS-FJD), Madrid, Spain*

^4^ *Department of Internal Medicine, Hospital Rey Juan Carlos, Madrid, Spain*

^5^ *Department of Epidemiology, Hospital Universitario Fundación Jiménez Díaz, Madrid, Spain*

^6^ *Department of Hematology, Hospital Universitario Fundación Jiménez Díaz, Madrid, Spain*

* Corresponding authors:

Fernando Sánchez-Vizcaíno: Address: Bristol Veterinary School, University of Bristol, Churchill Building, Langford Campus, Bristol, BS40 5DU, United Kingdom; E-mail address: [f.s-vizcaino@bristol.ac.uk](mailto:f.s-vizcaino@bristol.ac.uk); Tel: + 44 (0)117 928 9243

Juan Manuel Alonso-Domínguez: Address: Laboratorio Biología Molecular, Planta -1. Hospital Fundación Jiménez Díaz, Avda. Reyes Católicos, N 2, 28040 Madrid, Spain; E-mail address: [juan.adominguez@fjd.es](mailto:juan.adominguez@fjd.es); Tel: + 34 915504800 (extension 2673)

**Contents of this file**

Supplementary Methods

Supplementary Tables S1, S2 and S3

Supplementary Figure S1

**Supplementary Methods**

This section provides further details on the specification of the generalized linear autoregressive moving average (GLARMA) models used.

Briefly, the GLARMA models are summarized as follows. Let
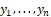
be the available observations on the discrete response series. Associated with these are vectors,
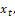
 of *K* regressors observed for
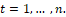
 Let
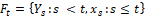
 denote the past information available on the response series and the past and present information on the regressors. This method models the distribution of the current observed number of AML diagnoses in month *t*,
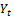
conditional on
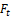
 as Poisson with conditional mean
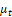
and density
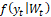
, with
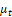
 given by


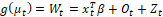
, (1)

where
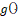
 is the canonical link function and
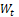
the canonical parameter or ‘state variable’. The vector
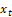
 denotes the set of explanatory variables observed at time *t* and
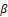
 their associated regression coefficients. The explanatory variables considered and their selection process were described in the main manuscript. An intercept was included by choosing the first column of the time-varying covariate matrix
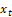
to be the vector
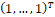
.
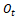
is an offset term which represents the logarithm of the Spanish population divided by 10^06 in each given year and month to provide a model for the incidence of AML per unit of million person-months. Serial correlation is induced by an autoregressive moving average (ARMA) structure of
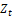
, which is given by


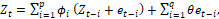
 (2)

where
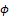
 and
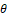
 are unknown parameters (in addition to the regression parameters
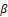
) for the autoregressive and for the moving average components, respectively. Pearson residuals were used as the predictive residuals, which are defined as


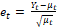
 (3)

The model order can be specified by considering the sets
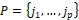
 and
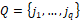
.

Appropriate lags for the AR and MA components were selected by starting with low orders for *p* and *q* and keeping them from having the same initial value. Once stability of estimation was achieved for a lower order specification, the values of *p* or *q* were progressively increased and coefficient estimates examined. For more information, the reader is referred to.^1^

References

1. Dunsmuir WTM, Scott DJ. The glarma package for observation-driven time series regression of counts. *J Stat Softw* 2015; **67**:1–36.

**Supplementary Table S1. Census of the Spanish general population and stratified by age and sex for years 2004-2015.**

| **Year** | **Age** | | | | | | | | | | | | | | | | | | | | |
| --- | --- | --- | --- | --- | --- | --- | --- | --- | --- | --- | --- | --- | --- | --- | --- | --- | --- | --- | --- | --- | --- |
|  | **0-4 years** | | | **5-19 years** | | | **20-49 years** | | | **50-64 years** | | | **65-74 years** | | | **>= 75 years** | | | **Total** | | |
|  | **Female** | **Male** | **Total** | **Female** | **Male** | **Total** | **Female** | **Male** | **Total** | **Female** | **Male** | **Total** | **Female** | **Male** | **Total** | **Female** | **Male** | **Total** | **Female** | **Male** | **Total** |
| 2004 | 957373 | 1017055 | 1974428 | 3182525 | 3358103 | 6540628 | 10001101 | 10404419 | 20405520 | 3549940 | 3426159 | 6976099 | 2131264 | 1811954 | 3943218 | 2090234 | 1267557 | 3357791 | 21912437 | 21285247 | 43197684 |
| 2005 | 1015288 | 1079294 | 2094582 | 3181772 | 3360222 | 6541994 | 10205059 | 10691791 | 20896850 | 3684262 | 3558575 | 7242837 | 2082212 | 1771760 | 3853972 | 2159068 | 1319227 | 3478295 | 21780869 | 22327661 | 44108530 |
| 2006 | 1055049 | 1119920 | 2174969 | 3181713 | 3363957 | 6545670 | 10309866 | 10833088 | 21142954 | 3742007 | 3618972 | 7360979 | 2092747 | 1793544 | 3886291 | 2227116 | 1370985 | 3598101 | 22100466 | 22608498 | 44708964 |
| 2007 | 1084747 | 1152780 | 2237527 | 3195375 | 3378807 | 6574182 | 10389390 | 10897179 | 21286569 | 3849405 | 3721228 | 7570633 | 2060277 | 1772940 | 3833217 | 2281581 | 1417028 | 3698609 | 22339962 | 22860775 | 45200737 |
| 2008 | 1133838 | 1205808 | 2339646 | 3241516 | 3429231 | 6670747 | 10561029 | 11126042 | 21687071 | 3979078 | 3848355 | 7827433 | 2041232 | 1764590 | 3805822 | 2353392 | 1473711 | 3827103 | 22847737 | 23310085 | 46157822 |
| 2009 | 1174754 | 1249291 | 2424045 | 3272531 | 3460485 | 6733016 | 10628817 | 11170235 | 21799052 | 4073428 | 3933362 | 8006790 | 2051986 | 1778555 | 3830541 | 2427303 | 1525060 | 3952363 | 23116988 | 23628819 | 46745807 |
| 2010 | 1194083 | 1269480 | 2463563 | 3293239 | 3482966 | 6776205 | 10590878 | 11081083 | 21671961 | 4158913 | 4019225 | 8178138 | 2068912 | 1800688 | 3869600 | 2488821 | 1572743 | 4061564 | 23226185 | 23794846 | 47021031 |
| 2011 | 1197021 | 1273539 | 2470560 | 3310593 | 3500929 | 6811522 | 10515657 | 10959038 | 21474695 | 4240092 | 4100067 | 8340159 | 2095773 | 1831101 | 3926874 | 2548170 | 1618513 | 4166683 | 23283187 | 23907306 | 47190493 |
| 2012 | 1184185 | 1259518 | 2443703 | 3328082 | 3518488 | 6846570 | 10417682 | 10826422 | 21244104 | 4325380 | 4183368 | 8508748 | 2104528 | 1847883 | 3952411 | 2607108 | 1662677 | 4269785 | 23298356 | 23966965 | 47265321 |
| 2013 | 1162081 | 1233651 | 2395732 | 3336305 | 3524916 | 6861221 | 10267609 | 10621434 | 20889043 | 4396866 | 4251060 | 8647926 | 2138753 | 1882137 | 4020890 | 2631783 | 1683188 | 4314971 | 23196386 | 23933397 | 47129783 |
| 2014 | 1117379 | 1184674 | 2302053 | 3352812 | 3542833 | 6895645 | 10051830 | 10356344 | 20408174 | 4434672 | 4288370 | 8723042 | 2200937 | 1938397 | 4139334 | 2628035 | 1675058 | 4303093 | 22985676 | 23785665 | 46771341 |
| 2015 | 1083014 | 1147833 | 2230847 | 3375921 | 3568212 | 6944133 | 9854787 | 10122844 | 19977631 | 4522564 | 4375222 | 8897786 | 2285593 | 2014244 | 4299837 | 2612120 | 1662028 | 4274148 | 22890383 | 23733999 | 46624382 |

**Supplementary Table S2.** Variance calculated for the observed time series and for its three additive components using STL.

| **Population** | **Variances** | | | |
| --- | --- | --- | --- | --- |
|  | **Trend** | **Seasonal** | **Remainder** | **Observed** |
| All | 0.015 | 0.034 | 0.136 | 0.188 |
| Female | 0.021 | 0.023 | 0.167 | 0.212 |
| Male | 0.015 | 0.066 | 0.315 | 0.40 |
| 0-4 years | 0.056 | 0.054 | 0.534 | 0.648 |
| 5-19 years | 0.013 | 0.013 | 0.132 | 0.159 |
| 20-49 years | 0.007 | 0.018 | 0.062 | 0.087 |
| 50-64 years | 0.022 | 0.116 | 0.805 | 0.975 |
| 65-74 years | 0.445 | 0.700 | 3.022 | 4.171 |
| ≥ 75 years | 2.15 | 0.83 | 5.544 | 8.562 |

**Supplementary Table S3.** Selected lags for the autoregressive and moving average components from the final Poisson GLARMA models.

| **Model** | **Autoregressive lags** | **Moving average lags** |
| --- | --- | --- |
| All cases | 3 | 7 |
| Female cases | .. | 23 |
| Male cases | 9 | 7 |
| Cases aged 0-4 years | .. | 24 |
| Cases aged 5-19 years | 8 | 4, 16 |
| Cases aged 20-49 years | 31 | 6 |
| Cases aged 50-64 years | 34 | .. |
| Cases aged 65-74 years | .. | 29, 33 |
| Cases aged 75 years and older | 7 | 10 |

**Supplementary Figure. S1.** Autocorrelation function (ACF) of the Pearson’s residuals from each final Poisson GLARMA model. Each ACF plot is generated from one of the nine final GLARMA models fitted to investigate the temporal dynamics in acute myeloid leukemia incidence for the overall population in Spain and stratified by sex and age from 2004 to 2015


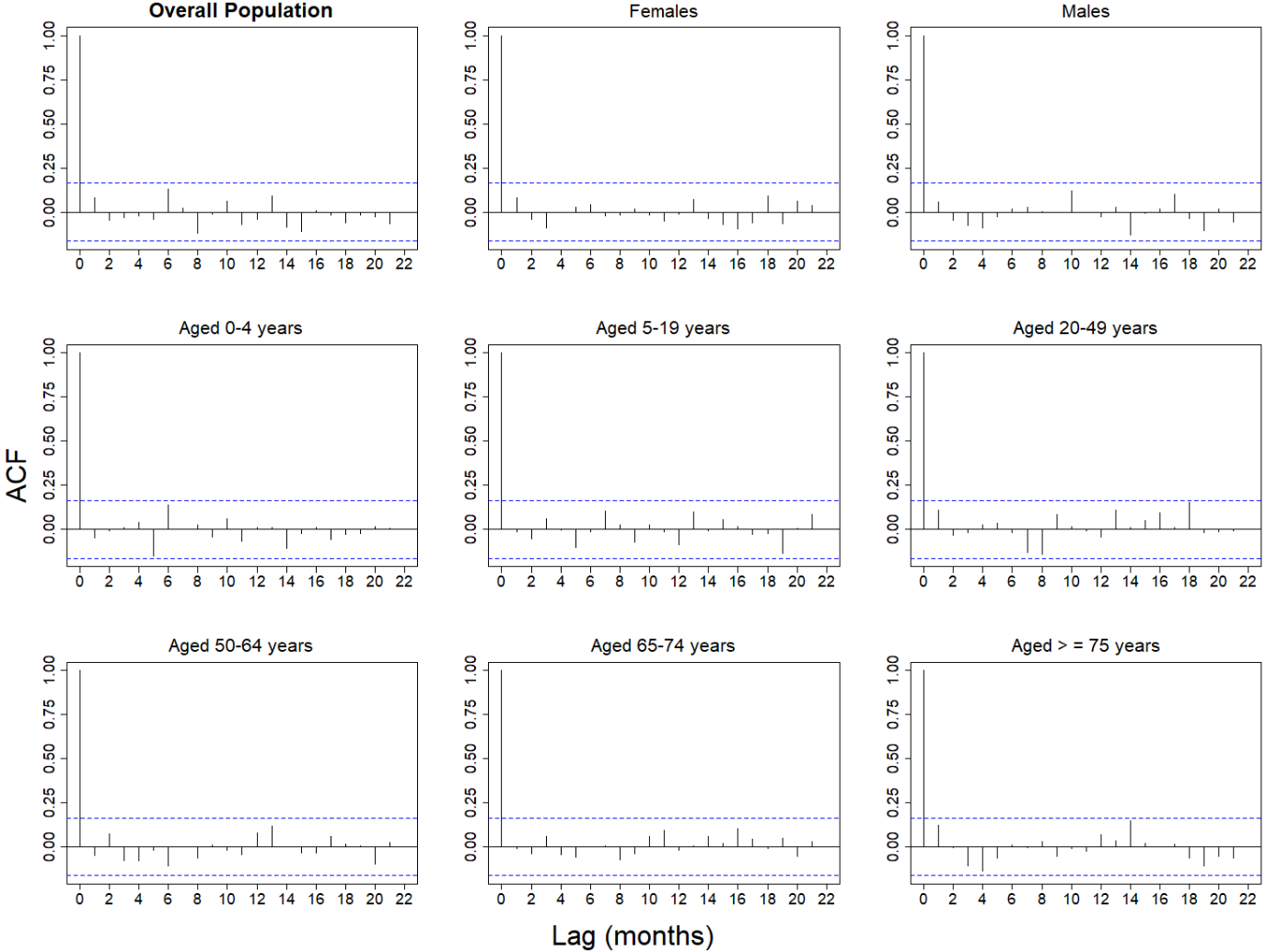

Supplement: Supplementary file 1 — Appendix S1 [file BJH-198-545-s001.doc]
